# Using individual growth model to analyze the change in quality of life from adolescence to adulthood

**DOI:** 10.1186/1477-7525-4-10

**Published:** 2006-02-21

**Authors:** Henian Chen, Patricia Cohen

**Affiliations:** 1 Epidemiology of Mental Disorders, New York State Psychiatric Institute, New York, NY, USA; 2 Department of Psychiatry, College of Physicians and Surgeons, Columbia University, New York, NY, USA; 3 Department of Epidemiology, Mailman School of Public Health, Columbia University, New York, NY, USA

## Abstract

**Background:**

The individual growth model is a relatively new statistical technique now widely used to examine the unique trajectories of individuals and groups in repeated measures data. This technique is increasingly used to analyze the changes over time in quality of life (QOL) data. This study examines the change from adolescence to adulthood in physical health as an aspect of QOL as an illustration of the use of this analytic method.

**Methods:**

Employing data from the Children in the Community (CIC) study, a prospective longitudinal investigation, physical health was assessed at mean ages 16, 22, and 33 in 752 persons born between 1965 and 1975.

**Results:**

The analyses using individual growth models show a linear decline in average physical health from age 10 to age 40. Males reported better physical health and declined less per year on average. Time-varying psychiatric disorders accounted for 8.6% of the explained variation in mean physical health, and 6.7% of the explained variation in linear change in physical health. Those with such a disorder reported lower mean physical health and a more rapid decline with age than those without a current psychiatric disorder. The use of SAS PROC MIXED, including syntax and interpretation of output are provided. Applications of these models including statistical assumptions, centering issues and cohort effects are discussed.

**Conclusion:**

This paper highlights the usefulness of the individual growth model in modeling longitudinal change in QOL variables.

## Background

Quality of life (QOL) has now become firmly established as an important and broad set of concerns in patient care and clinical research [1,2]. Improving QOL is a major goal in the treatment of individuals with medical disorders [3,4]. Many clinical trials now include patients' longitudinal QOL data [5-10]. Less is known about changes in QOL over time in the general population. Investigations of change in QOL in a given sample provide answers to two kinds of questions. First, what is the overall trend in QOL over time or age? Does the trajectory in a given sample increase, decrease, remain flat, or exhibit curvilinearity? Second, regardless of the shape and direction of the overall trajectory in QOL, are there individual differences surrounding it? If so, what variables are associated with differences in trajectories in QOL? Individual trajectories in QOL reflect within-person processes, whereas differences across trajectories reflect between-person differences. Individual growth models permit the integration of these two forms.

The individual growth model [11-16] is a relatively new statistical technique now widely used to examine the unique trajectories of individuals and groups in repeated measures data [17-20]. This technique is increasingly used to analyze the changes over time in QOL data [7-10]. This method overcomes some of the limitations of traditional repeated measure techniques and offers additional benefits and information. Repeated measure ANOVA requires balanced data with all individuals measured at each time point. It also assumes that the overall pattern of change within a sample generalizes to all individuals; individual differences in change are relegated to the bin of random error. An individual growth model estimates the average trajectory as well as individual trajectories, thus allowing for the explicit examination of inter-individual differences in intra-individual change. It readily estimates both linear and nonlinear change; it permits inclusion of individuals not assessed at all time points; and when age rather than secular time is the focus of the investigation allows data collected at a series of time-points from individuals from a range of birth cohorts to be combined in the analysis of age trajectories.

In this paper, we show how to use SAS PROC MIXED [12] to fit the individual growth models to QOL data from a community-based longitudinal study. First, we introduce the individual growth model, the QOL data used in this study and the specific longitudinal features we would like to examine. Second, we show how to fit an individual growth model by using SAS and how to interpret the results. Third, we estimate the overall trajectory of QOL as well as individual differences in the parameters that define this trajectory (e. g., slopes, intercepts). In addition, we attempt to account for such variability in trajectories by using gender and psychiatric disorder. Fourth, we discuss the application of individual growth models including statistical assumptions, centering issues and cohort effects.

## Methods

### Participants and study procedure

This study examined longitudinal data from the now-grown youths in the Children in the Community (CIC) study, an ongoing investigation of childhood behavior and development based on a sample of families randomly selected on the basis of residence in two upstate New York counties (21, 22). Approximately 800 mothers and one randomly sampled child from each family (mean age 5.5, SD = 2.8, in 1975) have been re-interviewed in their homes by extensively trained and supervised lay interviewers in 1985–1986 (n = 752), 1991–1994 (n = 751) and 2002–2004 (n = 641). These families were generally representative of the northeastern United States in terms of demographic characteristics and socioeconomic status (22). The sample also reflects the relatively high proportion of Catholic (54%) and Caucasian (91%) residents living in the sampled region. Detail of sampling, comparison to population, and retention rates are provided in the study website . The study procedures were approved in accordance with appropriate institutional guidelines by the Institutional Review Boards of the Columbia University College of Physicians and Surgeons and the New York State Psychiatric Institute. A National Institute of Health Certificate of Confidentiality has been obtained for these data. Written informed consent was obtained from all participants after the interview procedures were fully explained.

### Measures

#### Quality of life

**Figure 1:**
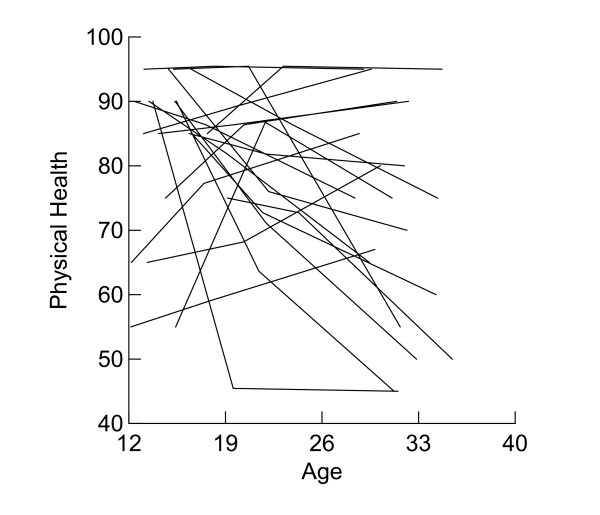
Individual Physical Health Change (raw data, n = 20).

Participating youth in 1985–86, 1991–94, and 2001–04 interviews completed the Quality of Life Instrument for Young Adults (YAQOL) [23-25]. The YAQOL is comprised of 14 multi-item scales that cover five domains of QOL of young adults (physical health, social relationships, psychological well-being, role function, and environment context). In the present study we use physical health data as an example. The physical health scale is composed of 8 items assessing overall health, incapacitation due to illness, and energy level. The measure is scaled so that the minimum possible score is defined as 0 and the maximum possible score as 100 with higher scores indicating better QOL. 580 subjects were assessed all three times (72.0%), 179 were assessed twice (22.2%) and 46 were assessed at a single age (5.7%). Internal consistency reliability of the physical health scale is 0.70 (1985–86), 0.71 (1991–94), and 0.76 (2001–04). Mean (SD) of physical health scores are 78.87 (13.13), 76.18 (14.97), and 67.43 (18.53) respectively. Skew of physical health scores are -0.95, -0.87, and -0.51 for the three waves of data. Kurtosis of physical health scores are 0.86, 0.88, and -0.06 respectively. In Figure 1, each line represents the physical health scores of 20 sampled individuals followed through the three waves. The graph illustrates the large inter-individual variability in physical health scores. As we can see, the physical health of some respondents increased from wave 3 to wave 5 although most decreased with age.

#### Psychiatric disorders

The parent and youth versions of the Diagnostic Interview Schedule for Children (DISC-I) [26] were administered to assess any psychiatric disorder (major depressive disorder, anxiety disorder and disruptive disorder). 19.4% (1985–86), 18.4% (1991–94), and 18.9% (2001–04) of the participants reported at least one of these psychiatric disorders.

#### Individual growth models

In longitudinal QOL data we have measures of QOL at multiple time points for each individual. Individual growth models allow us to use the trajectories of individuals across time or age as the basic unit of analysis. Trajectory aspects include mean over time or age: is an individual's average QOL score higher or lower than that of others? Does it rise or fall with age? Is change non-linear, such as declining gradually but then later plunging? In individual growth models, those questions represent the individual intercept, slope and quadratic slope. Individual growth models may estimate change trajectories over time measured as age at each assessment. In clinical samples time since illness or treatment onset is a common alternative. In the current illustration we "centered" age at 23 years, the age closest to the mean over the entire data set, by subtracting 23 from each participant's age at each assessment. Linear, quadratic, cubic or other models can be fit, as a function of age or time.

#### Setting up the data file

Virtually all programs that analyze growth or time-changing variables of individuals require that the basic file to be analyzed be set up such that each row represents a specific measurement time for a specific individual and each column a different variable. In this file some variables will be repeated unchanged for each participant, including that persons ID and gender. Other variables may change in each assessment, including the dependent variable, age, and possible time-varying predictors. There may be different numbers of assessments for different participants.

#### Unconditional growth model

For the unconditional linear growth model, the level-1 model is:

QOL_it _= α_i _+ β_i _+ r_it_

The level-1 model indicates each individual's standing on QOL as a function of his or her level of QOL at age 23 (α_i_), his or her linear growth trajectory (β_i_), plus his or her random error as it varies by age (r_it_). Level-1 models thus directly represent individuals' change trajectories.

The level-2 model is:

α_i _= G_00 _+ U_0i _and β_i _= G_10 _+ U_1i_

The level-2 model provides intercept and linear growth (slope over time) terms as the sample average, measured with some error. In addition to the average of the intercept and slope (fixed effects), the variances of the intercept and slope (random effects) are also obtained. It is important to note that even if the average slope is not significantly different from zero, significant variability in slope associated with the time variable in the level-1 model indicates that individuals are changing in QOL, although in different directions.

#### Conditional growth model

Once the unconditional linear growth model was selected for our QOL data, we may further determine whether the intercepts and linear slopes vary as a function of differences between the participants. The level-2 model may be expanded to become a "conditional" model. As in ordinary linear regression, additional predictors may be included in subsequent models. If those measures are constant across the time/age points they are considered "fixed" predictors (e.g., gender). If they also may change over the multiple assessments they are considered "time-varying predictors (e.g., psychiatric disorder). In either case such variables are added to the level 2 model to determine their association with QOL and the extent to which they may account for a fraction of the sample mean or linear trajectory. For example, with gender in the level-2 model:

α_i _= G_11 _+ G_12 _(gender) + U_1i_and β_i _= G_21 _+ G_22 _(gender) + U_2i_

We coded female 0 and male 1 in our data. In the conditional level-2 model, G_11 _and G_21 _represent the average intercept at age 23 and linear slope for female. G_12 _and G_22 _represent the mean difference between men and women for the average intercept at age 23 and linear slope.

### Fitting individual growth models using SAS

#### Unconditional growth model (basic growth model)

We can fit the unconditional growth model in SAS PROC MIXED (12) quite easily using the following syntax:

proc mixed noclprint covtest noitprint;

class id;

model health = age/solution ddfm = bw notest;

random intercept age/subject = id;

run;

The PROC MIXED statement calls the procedure. NOCLPRINT prevents printing the CLASS level information. COVEST tests the variance and covariance components (random effects). NOITPRINT statement tells SAS not to print the iteration history. The CLASS variable specifies that ID is a classification variable to indicate that the data represents multiple observations over time for individuals. MODEL statement is an equation whose left-side contains the name of the dependent variable, in this case HEALTH. The right-hand side contains a list of the fixed-effect variables (predictors). The intercept is contained in all models. This unconditional model tests only the intercept and slope without any predictors. DDFM = BW asks SAS to use the "Between/Within" method for computing the denominator degrees of freedom for tests of the fixed effects. NOTEST prevents the printing results of type 3 tests of fixed effects. RANDOM statement contains a list of the random effects, in this case intercept and age.

#### Conditional growth model for gender

Based on the unconditional growth model, we can add gender into the model and test the mean and slope differences in physical health by gender. The SAS syntax is:

proc mixed noclprint covtest noitprint;

class id;

model health = age gender gender*age/solution ddfm = bw notest;

random intercept age/subject = id;

run;

The only change in this model is adding gender and gender*age in the right-hand side of the MODEL statement as predictors.

#### Conditional growth model for psychiatric disorders

Based on the conditional growth model for gender, we add a time-varying variable reflecting the presence of a psychiatric disorder and its product with age into the model and test the mean and slope differences in physical health associated with psychiatric disorder in a model that includes gender and age-gender product. The SAS syntax is:

proc mixed noclprint covtest noitprint;

class id;

model health = age gender gender*age disorder disorder*age/solution ddfm = bw notest;

random intercept age/subject = id;

run;

## Results

### Unconditional linear growth model

Table 1 presents the results of fitting the unconditional linear growth model. The estimated variance of intercepts and slopes is 101.53 (P < 0.001) and 0.30 (p < 0.001) respectively. The significant intercept variance means that individuals varied in the level of physical health; the significant slope variance indicates that they varied in rate and direction of change in physical health. The average young adult had a physical health score of 74.71 at age 23, and this decreased about 0.63 percentage points (PP) per year from age 10 to age 40.

**Table 1:** Individual growth models for longitudinal changes in physical health^a^

	**Unconditional Linear Model**	**Unconditional Non-linear Model**	**Gender**	**Psychiatric Disorder**
	Estimate (SE)	Estimate (SE)	Estimate (SE)	Estimate (SE)
Random Variance				
Intercept	101.53 (8.14) ***	101.68 (8.11) ***	87.34 (7.39) ***	79.86 (7.09) ***
Linear Slope	0.30 (0.08) ***	0.31 (0.08) ***	0.30 (0.08) ***	0.28 (0.08) ***
Residual	130.45 (7.17) ***	129.36 (7.12) ***	128.50 (6.96) ***	128.71 (7.04) ***
				
Fixed Effects				
Intercept	74.71 (0.44) ***	75.19 (0.52) ***	70.95 (0.59) ***	72.26 (0.60) ***
Age	-0.63 (0.04) ***	-0.59 (0.05) ***	-0.73 (0.06) ***	-0.67 (0.06) ***
Age^2^		-0.01 (0.01)	--	--
Gender			7.61 (0.84) ***	7.24 (0.81) ***
Gender × Age			0.25 (0.08) **	0.22 (0.08) **
Psychiatric Disorder				-5.95 (0.87) ***
Psychiatric Disorder × Age				-0.23 (0.11) *
				
Goodness of Fit^b^				
Parameters	5	6	7	9
Raw Likelihood (-2LL)	17624.0	17627.0	17538.3	17485.8
X^2^		3.0	85.7 ***	138.2***
Degrees of Freedom		1	2	4

Note. SE = standard error; LL = log likelihood.

^a^All parameter entries are maximum likelihood estimates fitted using SAS PROC MIXED.

Age was centered at 23 years, Gender was coded 0 = Female, 1 = Male.

Psychiatric disorder was coded 0 = no disorder, 1= disorder.

^b^Models for non-linear, gender and psychiatric disorder are compared with the unconditional linear growth model.

* p < 0.05; ** p < 0.01; *** p < 0.001

### Unconditional non-linear growth model

We add age*age (quadratic age) in the unconditional linear growth model to test the non-linear change in physical health. There was a non-significant negative quadratic age change in physical health (p = 0.08). The unconditional non-linear growth model was not significantly improved compared to the unconditional linear growth model (X^2 ^= 3.0, df = 1, p > 0.05). Therefore, we used the unconditional linear growth model as our basic growth model.

### Conditional growth model for gender

**Figure 2:**
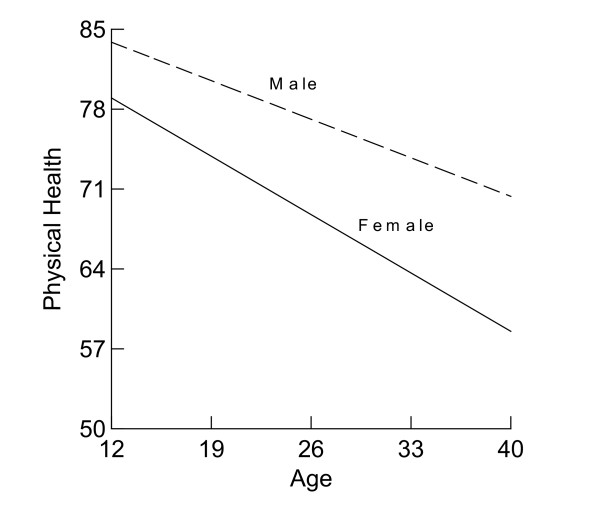
Physical Health Change by Gender.

Gender was powerful predictor of level of physical health [23], but was gender also an influential predictor of rate of change in physical health? As can be seen in Table 1, the variance for the intercepts changed from 101.53 to 87.34. Computing (101.53 – 87.34)/101.53 = 0.140, we find a 14.0% reduction. In other word, gender and its interaction with age accounted for 14.0% of the individual differences in mean physical health. The variance in linear slope did not change. Men reported 7.61 PP higher mean physical health than did women. The significant interaction between age and gender indicates that the annual decline in physical health by women of 0.73 PP was significantly greater than the annual decline in physical health by men (-0.73 + 0.25 = -0.48) (Figure 2).

### Conditional growth model for psychiatric disorders

**Figure 3:**
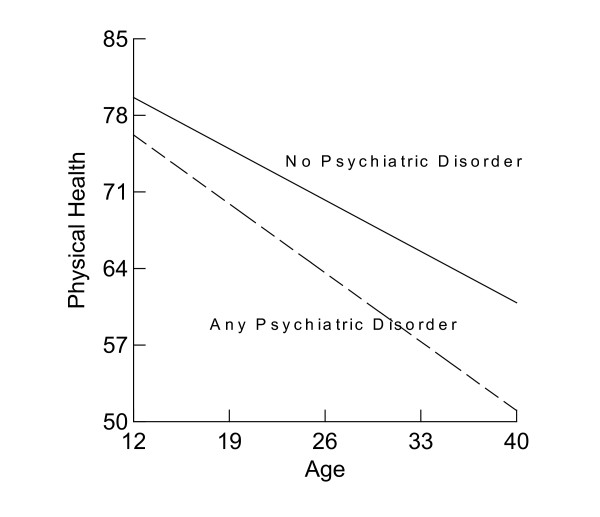
Physical Health Change by Psychiatric Disorder.

Psychiatric disorders have been associated with a lower level of physical health [24,25], but it is not clear whether psychiatric disorder is also an influential predictor of rate of change in physical health. Compared with the model for gender, the variance among participants' mean physical health declined from 87.34 to 79.86 or about 8.6% (Table 1). The unexplained individual variance in annual change diminished only slightly from 0.30 to 0.28. Study participants with a psychiatric disorder reported 5.95 PP lower mean physical health and a 0.23 PP faster decline per year on physical health from age 10 to age 40 than those without any psychiatric disorder net of gender differences (Figure 3).

## Discussion

Individual growth models are increasingly used to analyze the change in QOL data over time as more clinical trials include patients' longitudinal QOL data now [5-10]. Traditional models such as repeated measure ANOVA are not readily used for these analyses because the standard requirements of equal numbers and intervals of assessment are typically not met. The subsequent potentially substantial loss of information may result not only in a lowering of statistical power but also in a potentially biased subsample used in the final analyses. Although individual growth models have been discussed for a number of years in education and other disciplines [11,13,14,17-20], they have only recently been gaining attention in the QOL field [7-10]. Although such models have important limits, they represent a substantial technical advance.

As noted in a basic regression text [16], the slope parameter represents the average increase in the dependent variable for a unit increase in the predictor variable, while the intercept parameter represents the expected value of the outcome measure when all the predictors are zero. In our data, the intercept term represents the predicted level of QOL for a person at his or her age 23, coded 0 here in order to keep the estimated mean at an age actually included in the study. Such "centering" by subtracting the average time of assessment makes the intercept more interpretable. It also eliminates the correlation of the average linear change over time with a squared age variable which may be used to identify a curvilinear average change over time. In general, centering is also helpful for all (non-dichotomous) predictor variables for which effects may depend on (vary with the value of) some other predictor variable. Several researchers have discussed the centering issues in individual growth models [16,27,28]. In these analyses, we begin with the assumption that the QOL score may change be linearly with age. We also assume that the change in QOL does not differ as a function of the individual's age at the first occasion of measurement, which would require adding age1 as a predictor in the model to test the cohort effects. In a clinical sample with a large age range at the first occasion of measurement, age1 would need to be included in the model.

The statistical maximum likelihood model used to generate these estimated effects assumes multivariate normality of the model residuals, linear relationships, and homoscedasticity. When the dependent variable distribution is seriously non-normal this assumption may be violated and a transform of the original dependent variable to more nearly normal distribution is likely to be necessary [16]. The interested reader is referred to the helpful papers by Maas & Hox [29,30] for the consequences of the violation of this assumption. Another solution is to use, generalized estimating equations (GEE) [31], an alternative method that is (in our experience, slightly) more robust to this assumption failure. A disadvantage of GEE for estimating longitudinal change is that GEE does not estimate the random effects, which are informative about the amount of variance among sample members that is attributable to predictor variables.

We fit a growth model for our QOL data in which both intercepts and slopes vary across persons. We did not explore the within-person error covariance structure because these data consisted of only three longitudinal time points. With additional observations per person, additional structures for the within-person error covariance are possible. Three of the most commonly used structures are compound symmetry, unstructured, and autoregressive order one. The structure of the within-person error covariance matrix is specified using a REPEATED statement in SAS. The interested reader is referred to the SAS PROC MIXED (12), the helpful paper by Wolfinger (32) and the book by Singer and Willett (33).

## Conclusion

This paper highlights the utility of growth model analyses in modeling longitudinal change in QOL variables.

## Authors' contributions

Patricia Cohen and Henian Chen were responsible for conceptualization and design of the study and quality of life data collection. Henian Chen analyzed the data, interpreted the findings, and drafted the article. Patricia Cohen supervised the data analysis and assisted with the interpretation of findings and the critical revision of the article. Both read and approved the final manuscript.
